# Evolutionary conservation of DNA-contact residues in DNA-binding domains

**DOI:** 10.1186/1471-2105-9-S6-S3

**Published:** 2008-05-28

**Authors:** Yao-Lin Chang, Huai-Kuang Tsai, Cheng-Yan Kao, Yung-Chian Chen, Yuh-Jyh Hu, Jinn-Moon Yang

**Affiliations:** 1 Department of Computer Science and Information Engineering, National Taiwan University, Taipei 106, Taiwan; 2 Institute of Information Science, Academia Sinica, Taipei 115, Taiwan; 3 Institute for Information Industry, Taipei 106, Taiwan; 4 Institute of Bioinformatics, National Chiao Tung University, Hsinchu 30050, Taiwan; 5 Department of Computer Science, National Chiao Tung University, Hsinchu 30050, Taiwan; 6 Department of Biological Science and Technology, National Chiao Tung University, Hsinchu 30050, Taiwan; 7 Core Facility for Structural Bioinformatics, National Chiao Tung University, Hsinchu, Taiwan

## Abstract

**Background:**

DNA-binding proteins are of utmost importance to gene regulation. The identification of DNA-binding domains is useful for understanding the regulation mechanisms of DNA-binding proteins. In this study, we proposed a method to determine whether a domain or a protein can has DNA binding capability by considering evolutionary conservation of DNA-binding residues.

**Results:**

Our method achieves high precision and recall for 66 families of DNA-binding domains, with a false positive rate less than 5% for 250 non-DNA-binding proteins. In addition, experimental results show that our method is able to identify the different DNA-binding behaviors of proteins in the same SCOP family based on the use of evolutionary conservation of DNA-contact residues.

**Conclusion:**

This study shows the conservation of DNA-contact residues in DNA-binding domains. We conclude that the members in the same subfamily bind DNA specifically and the members in different subfamilies often recognize different DNA targets. Additionally, we observe the co-evolution of DNA-contact residues and interacting DNA base-pairs.

## Background

DNA-binding proteins play a key role in living organisms of many genetic activities such as transcription, recombination, DNA replication and repair. One or more domains of these proteins interact with DNA, and they offer the specificity for direct and indirect readout of DNA [1]. To identify the DNA-binding domains is very important for understanding the regulation mechanisms.

Recently, rapidly increasing amount of protein-DNA complexes from X-ray crystallography and nuclear magnetic resonance (NMR) have enabled the use of structural-based approaches for identifying DNA-binding proteins. Most of the structural DNA-binding domains can be categorized into several classes according to their structures or binding types [2-4]. However, some DNA-binding domains can not be well categorized, and for some DNA-binding domains structural information is unavailable [3,5]. Several studies used various computational approaches to predict potential DNA-binding proteins by using protein-DNA complexes structure features, such as the overall charges, electric moments, and shape of binding sites [6-12]. Since the charge and conformational complementarities of binding sites are essential for protein-DNA binding, these features provide a reasonable basis to identify DNA-binding proteins. Another trend is to consider the degree of conservation of residues [13-15]. Luscombe and Thornton [16] have studied 21 families of DNA-binding proteins and showed that those amino acids interacting with the DNA are better conserved than those not interacting with DNA. Stawiski et al. [17] found that electrostatic patches of DNA-binding proteins have a higher percentage of aromatic and positive residues. According to the general properties of 20 amino acids, they also showed that residues of the patch are conserved at property levels.

In this paper, we propose a structure-based threading method by considering evolutionary conservation of DNA-contact residues in DNA-binding domains to identify DNA-binding domains. We use BLOSUM62 [18], an evolutionary-based scoring matrix for amino acid substitutions, to measure the degree of conservation of binding residues. Our method can achieve high precision and recall for 66 families of DNA-binding domains, with a false positive rate less than 5% for 250 non-DNA-binding proteins.

## Results

Given a query domain, our method identified similar DNA-binding structures or homologous protein sequences from the template library. To evaluate the performance of our method, for each DNA-contact domain (*D*) in the template library we generated its corresponding positive and negative sets. The members in the positive set contain the domains similar to domain *D *based on SCOP, while domains in the negative set do not. By applying our method on these two sets, we found that the scores of the domains in the positive set are significantly higher than those of domains in the negative set. We further determined a threshold to achieve high precision and recall. Combining with the threshold, we applied our method on 66 known SCOP families of DNA-binding domains and 250 non-DNA-binding proteins to examine the performance.

### Positive and negative set for each contact domain

We collected DNA-binding contact domains from SCOP database, the detail is described in Method. To remove redundant contact domains, domains with highly similar sequences (identity > 90%) are grouped using the NCBI software BLASTCLUST. In each group, the one with the maximal number of contact residues is chosen as the representative domain of a group. For a representative domain *R*, these protein domains in the same SCOP family are considered as the member of *R *according to SCOP95 (members whose similarity greater than 95% are excluded). Each member of *R *was aligned to *R *using the CE. We define a residue of *R *as misaligned if it is aligned to a gap. A family member is discarded if more than 20% contact residues of *R *are misaligned between *R *and this member. Family members that satisfy the above criteria are considered to be in the positive set. If there are less than five members in the positive set of *R*, the entire family of *R *is discarded. We finally yielded 66 representative domains with corresponding positive sets. For each *R*, we artificially generated 1000 domains to be the negative set. To do this, for each artificial domain, we replicate its residues from *R*. Then we randomly mutated the residue type of each contact residue of *R*.

### Determining the threshold of similar DNA-binding function of a contact domain

For each representative domain *R*, each member in the positive and negative sets was scored by the method we developed. Ideally, the scores of domains in the positive set should be on average significantly higher than those of the negative set. We used the Kolmogorov-Smirnov (KS) test to examine the above criterion. The KS test is a nonparametric test to determine if two distributions differ significantly. According to our results, the scores are significantly different for the positive set and the negative set in most domains (97% of 66 sets have a *p *value less than 0.05).

**Figure 1:**
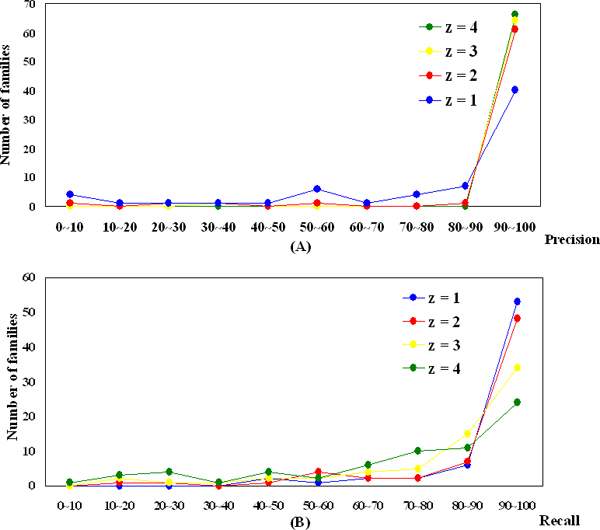
**Precision and recall on different z-score thresholds**. Our method results on different z-score thresholds for 66 representative domains. The distributions of the numbers of the families for (A) precisions and (B) recalls.

Further, given a contact domain, we would like to determine a threshold for determining which domains have a similar DNA-binding function. For the two sets (positive and negative) of a representative domain, we separately transform all members' scores to z-scores by

$$z=\frac{s-\mu }{\delta },$$

where *s *is the score of a member, *μ *is the mean score of the these two sets, and *δ *is the standard deviation. Figures 1A and 1B show the precision (ratio of the number of retrieved true positive data to all retrieved data) and the recall (ratio of the number of retrieved true positive data to all true positive) with various z-score thresholds, respectively. As shown in Figure 1A, when we set the threshold greater than two, the precisions of using different thresholds are very similar (>90%). If we set the z-score threshold to one, only 60% of families are with high precision. The results imply that larger thresholds will yield higher precisions, but the benefit is limited when the threshold is larger than two. Oppositely, as shown in Figure 1B, larger thresholds will reduce the recall. According to these results, we take the z-score threshold as 2.0 and the domains with a z-score higher than the threshold will be considered as putative DNA-binding domains.

### Non-DNA-binding proteins

**Figure 2:**
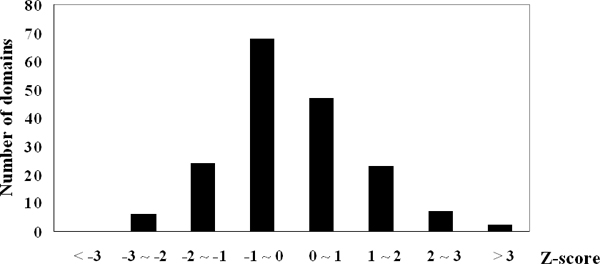
Distribution of z-score values of 177 non-DNA-binding domains.

We further apply our method to 250 non-nucleic-acid binding (non-DNA-binding) proteins, which were initially studied by Hobohm and Sander [19] and further specified by Stawiski *et al. *[17]. We align all non-redundant contact domains to those non-DNA-binding proteins using CE. Alignments whose z-scores (defined by CE) are greater than 3.7 with the misalign rate of contact residues less than 20% are chosen as non-DNA-binding domains. 177 non-DNA-binding domains pass the constraints among 250 proteins. We applied our method on these non-DNA-binding domains and transformed their scores to *z*-scores. Figure 2 shows the distribution of z-scores of non-DNA-binding domains. The scores approximately follow a normal distribution and the peak of the density occurred at *Z *= -1~0. Given a z-score threshold, the false positive rate is the ratio of number of domains whose z-score are beyond the threshold to the total non-DNA-binding domains. According to our previous analysis, we set the threshold to 2.0 and the false positive rate is less than 0.05. It shows that for non-DNA-binding domains, our method can recognize their non-binding with high accuracy.

## Discussion

**Figure 3:**
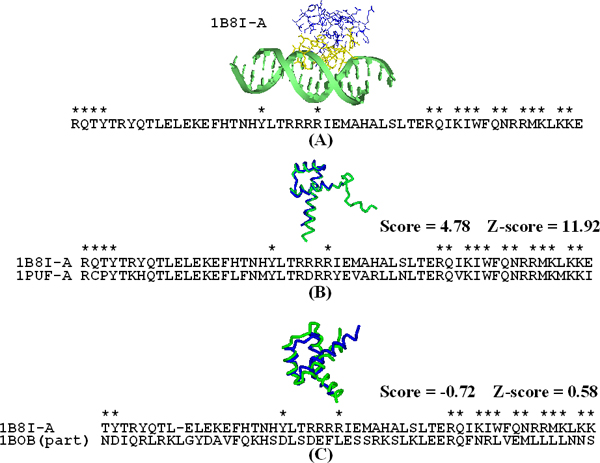
**Searching results of the ultrabithorax homeodomain protein**. Searching results using the homeotic Ubx/Exd/DNA ternary complex (PDB entry 1B8I-A) from *Drosophila melanogaster *as the query. **(A) **The contact residues of 1B8I-A complex are presented as stick (yellow). The sequence of 1B8I-A is shown and contact residues are marked with asterisks. **(B) **Structure alignment of 1B8I-A (blue) and 1PUF-A (green). The score is 4.78 and Z-score is 11.92 by our scoring method. **(C) **Structure alignment of 1B8I-A (blue) and non-DNA-binding protein 1BOB (green). Only the aligned structure/sequence of 1B8I-A and 1BOB are shown. We obtained score = -0.72 and *Z*-score = 0.58.

Figure 3A shows an example, which is the ultrabithorax homeodomain (Ubx) from *Drosophila melanogaster *(PDB entry 1B8I-A[20]) selected from 66 representative domains to described the characteristics of our method. The DNA is represented in green. 18 DNA-contact residues are presented as yellow stick and other residues are denoted as blue. The protein sequence is also presented and a contact residue is marked with an asterisk. For the alignment of the representative domain (1B8I-A) to the domains of its member, Figure 3B presents a nice case (PDB entry 1PUF-A), which is a homeobox protein hox-a9 from mouse [21]. We found that the contact residues is highly conserved in the aligned amino acids of the two domains and our scoring method shows this high z-score (z-score = 11.92). On the other hand, if we align 1B8I-A to 250 non-DNA-binding proteins, our method is able to discard the similar protein structures whose contact residues are not conserved (z-score = 0.58). Figure 3C shows an example of aligning 1B8I-A to 1BOB, which is histone acetyltransferase hat1 from *S. cerevisiae *in complex with acetyl coenzyme [22].

**Figure 4:**
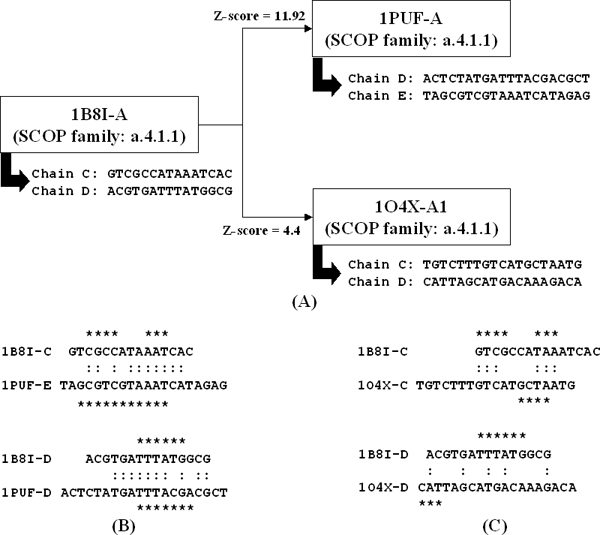
**Comparison of bound DNA sequences of homologous proteins**. The alignments of the bound DNA sequences of homologous proteins by using the homeotic ubx/exd/DNA ternary complex (PDB entry 1B8I-A) as the query. **(A) **The z-score values and the bound DNA sequences of the complex 1B8I (PDB entry 1B8I-C and 1B8I-D), 1PUF (PDB entry 1PUF-D and 1PUF-E), and 1O4X (PDB entry 1O4X-C and 1O4X-D). All sequences are from 5' to 3'. **(B) **Alignments of bound DNA sequences of the complexes 1B8I and 1PUF. A colon denotes an identical pair and an asterisk denotes a contact nucleotide (asterisks are marked above/below alphabets on the upper/lower sequence of the alignment, respectively). **(C) **Alignments of bound DNA sequences of the complexes 1B8I and 1O4X.

The z-score of DNA-binding domains in the same SCOP family may be variable for several representative domains (Figure 4A). The 1PUF-A and 1O4X-A1 (Oct-1 POU homeodomains from *Homo sapiens *[23]) are the members of the 1B8I-A representative domain. The *z*-scores are 11.92 (1PUF-A) and 4.4 (1O4X-A1) when 1B8I-A was used as the query (Figure 4A). The z-scores indicated that the contact residues between 1PUF-A and 1B8I-A are more conserved than the ones between 1O4X-A1 and 1B8I-A on contact residues interacting to the bases of the core binding site in the DNA.

To investigate variation of contact residues of DNA-binding domain in the same SCOP family, we compared the bound DNA sequences of two DNA-binding domains by aligning the double-strand sequences to each other. 1B8I-A binds two DNA sequences (i.e. PDB entry 1B8I-C and 1B8I-D) and 1O4X-A1 binds another two DNA sequences (PDB entry 1O4X-C and 1O4X-D). First we generated four pairing alignments: 1B8I-C and 1O4X-C; 1B8I-C and 1O4X-D; 1B8I-D and 1O4X-C; and 1B8I-D and 1O4X-D. We do not allow any gap insertion when aligning a-pairing DNA sequences. The alignments are obtained by sliding two sequences against each other until the best match is found. The alignment with the maximum number of identical aligned pairs is chosen, and as a result the alignment between 1B8I-C and 1O4X-C is the one chosen (Figure 4C). Then we adjust the alignment of the other DNA strand pairs (i.e. 1B8I-D and 1O4X-D) according to this best alignment (1B8I-C and 1O4X-C).

Figures 4B and 4C show that the number of identical nucleotides between 1B8I-C and 1PUF-E (10) as well as 1B8I-D and 1PUF-D (10) is much higher than those of 1B8I-C and 1O4X-C (6) as well as 1B8I-D and 1O4X-D (5) for whole DNA sequences. At the same time, 11 identical contact nucleotides are obtained from the alignments of 1B8I-C and 1PUF-E as well as 1B8I-D and 1PUF-D; but two identical contact nucleotides are yielded from the alignments of 1B8I-C and 1O4X-C as well as 1B8I-D and 1O4X-D (the contact nucleotides are the nucleotides that interact with contact residues of protein). With respect to 1B8I-A, 1PUF-A and 1O4X-A1 are different not only in the DNA sequences they bind to but also in their DNA-binding sites. These results show that the members in the same SCOP family may have different DNA-binding models and that our method is able to detect the different Protein-DNA interactions based on the evolutionary conservation of DNA-contact residues.

**Figure 5:**
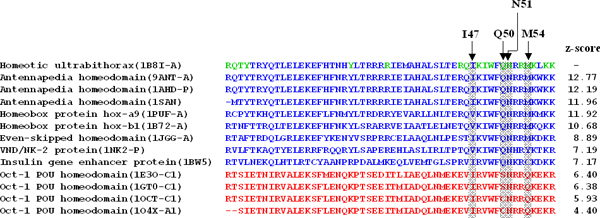
**Multiple structure alignment of 13 homeodomain structures**. The domains with similar DNA-binding specificities with 1B8I-A are shown in blue and others are red. The contact residues of 1B8I-A are marked green. The contact residues interacting to the bases of the core binding site in the DNA (ATAA) major groove are indicated gray.

We produced multiple protein sequence alignments of 13 homeodomains (Figure 5) selected from SCOP 1.71 using a multiple structure alignment tool, MUSTANG [24]. These domains were ranked by z-scores calculated by using our scoring method and the sequence of 1B8I-A as the query. According to z-scores, these 13 domains can be roughly divided into two groups, including the Ubx-like homeodomain colored in blue (e.g. PDB entry 9ANT-A (12.77), 1AHD-P (12.19), and 1SAN (11.96)) and the Oct-1 POU homeodomain colored in red (e.g. PDB entry 1E3O-C1 (6.40), 1GT0-C1 (6.38), and 1O4X-A1 (4.40)). Figure 5 shows that all Ubx-like homeodomains are significantly more conserved than Oct-1 POU homeodomains on contact residues (green). The Ubx homeodomain binds together with the extradenticle homeodomain (Exd) to recognize four DNA bases (ATAA) [20] based on four residues that are Ile47, Gln50, Asn51, and Met54, locating at *α*3 helix in the Ubx (gray columns in Figure 5). The z-scores of the domains are higher if they are conserved on these four residues, such as three antennapedia homeodomains and two homeobox protein hox. These results show that contact residues interacting with bases in the DNA sequences are often conserved. This result is consistent to previous results [16] in which the homeodomain family was considered as a multi-specific family that consists of some subfamilies. This work concluded that members in the same subfamily bind DNA specifically but the members in different subfamilies recognize different DNA targets. In summary, we demonstrated the conservation of DNA-contact residues in DNA-binding domains.

## Conclusion

The contact residues of DNA-binding domains are useful in discriminating DNA-binding domains from non-DNA-binding domains in a novel protein sequence. Our method, which considers evolutionary conservation of DNA-binding residues, can achieve high precision and recall for 66 families of DNA-binding domains, with a false positive rate less than 5% for 250 non-DNA-binding proteins. In addition, our method is able to identify the different DNA-binding behaviors of proteins in the same SCOP family based on the evolutionary conservation of DNA-contact residues. We also discussed the mutation of contact residues of DNA-binding domains can possibly change the bound DNA sequences. It implies that the co-change of DNA-contact residues and their DNA-binding bases.

## Methods

**Figure 6:**
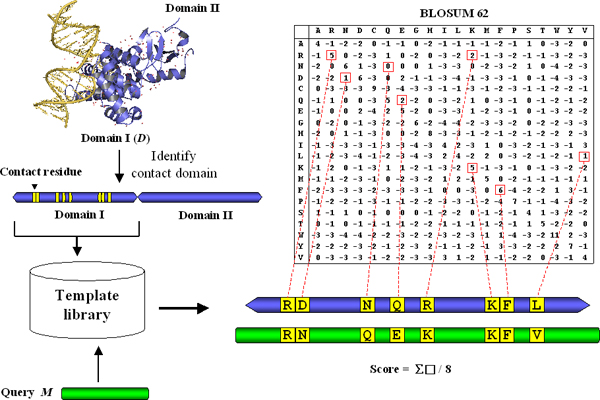
**Flowchart of proposed method**. See text.

Figure 6 shows the flowchart of our proposed method. We quantitatively evaluated whether a given protein domain *M *has a similar DNA-binding function to a known crystal protein-DNA structure. For each crystal structure of protein-DNA complex in Protein Data Bank (PDB), we first identified the DNA-contact domain (*D*) using geometry information and domain definitions from Structure Classification of Proteins (SCOP, version 1.71) [25]. The structures and sequences of both protein-DNA complexes and their DNA-contact domains were collected in the template library. For a given protein sequence/structure *M*, we used sequence/structural alignment tools to find the homologous DNA-contact domain *D *from the template library. Finally, we proposed a score method to evaluate the similarity between *M *and *D *based on the BLOSUM matrix. Detailed descriptions are as follows.

### Template library

We first collected protein-DNA complexes from PDB and each complex should contain at least one protein chain and a double-strand DNA. As in Luscombe et al. [26], a complex was excluded if its DNA is single-stranded or the length of the DNA is less than 4 bases. For each protein-DNA complex, we then identify contact residues and contact domains of this protein. Contact residues, whose heavy atoms are within a distance (distance ≤ 4.5 Å) of any heavy atoms of the bound DNA, are considered as the core parts of the contact domain in a complex [27]. For each protein-DNA complex, we identified its DNA-contact domains according to contact residues and the definition of the SCOP database. Each domain must have more than 5 contact residues and the number of residues of this protein is more than 50 to make sure that the contact between the protein and DNA was reasonably extensive. Finally, 230 contact DNA-binding domains were identified and collected in the template library.

### Homologous proteins searching

For a given protein sequence/structure *M*, we found a homologous DNA-binding protein from the template library using alignment tools. If *M *is a 3D-structure, we used a structure alignment (i.e. CE [28]) to align *M *to all contact domains. The CE will return a *Z *score for each alignment representing the structure similarity of the two aligned structures. DNA-binding proteins are considered as homologous proteins of query *M *if CE *Z *scores of exceed 3.7 based on CE's statistical model. On the other hand, if *M *is a protein sequence, we used sequence alignment (i.e. FASTA [29-31]) to search the template library. Here, a DNA-binding protein is considered a homologous protein of *M *if the sequence identity exceeds 25% according to observations of previous studies [32-37].

### Scoring method

For an alignment of the query domain (*M*) and a contact domain (*D*) that satisfies the above criterion, we calculate the alignment score for the aligned contact residues by using the BLOSUM62 matrix. The scoring method is defined as:

$$S_{M}=\frac{\sum_{i\in CR}BLOSUM62(d_{i},m_{i})}{\text{\#contact residues}},$$

where *CR *is the set of the contact residues between *D *and *M*; *d*_*i *_and *m*_*i *_denote the corresponding *i*^*th *^contact residue of *D *and *M*, respectively. Here, the score of a misaligned residue is -4 which is the smallest in the BLOSUM62 matrix.

Authors' contributionsYLC and HKT carried out the design of scoring functions and data set preparation, participated in experimental designs and drafted the manuscript. CYK provided the design of this study. YCC and YJH provided the domain knowledge and useful comments. JMY provided the original idea, participated in the design and coordination of this study and helped to draft the manuscript. All authors read and approved the final manuscript.

## Competing interests

The authors declare that they have no competing interests.
